# ﻿First immature of the New World treehopper *Campylocentrusnigris* Funkhouser, 1930 (Hemiptera, Membracidae, Centrotinae, Boocerini) and biological notes from Costa Rica

**DOI:** 10.3897/zookeys.1249.152868

**Published:** 2025-08-19

**Authors:** Andrey J. Peraza-Sánchez, Stuart H. McKamey

**Affiliations:** 1 Universidad Estatal a Distancia (UNED), Alajuela, Costa Rica Universidad Estatal a Distancia Alajuela Costa Rica; 2 Systematic Entomology Laboratory, Agricultural Research Service, U.S. Department of Agriculture, c/o National Museum of Natural History, P.O. Box 37012, Washington, D.C. 20013, USA c/o National Museum of Natural History Washington, D.C. United States of America

**Keywords:** Behavior, distribution, host plant, immature stage, *Sicyosedulis*

## Abstract

The last immature stage of the New World treehopper *Campylocentrusnigris* Funkhouser, 1930 is described for the first time. Detailed figures of the adult are also provided, as well as new records, data on its behavior, distribution for Costa Rica, and host plants. Its host plant, chayote, is an important food staple locally and also a U.S. import. Panama is a new country record for the species.

## ﻿Introduction

Adult treehoppers are widely recognized for their remarkable and often highly elaborate pronotum, a distinctive feature that is prevalent across almost all of the over 400 genera and approximately 3,000 described species (McKamey 1998). The subfamily Centrotinae is the largest and only cosmopolitan subfamily of the treehopper family Membracidae, comprising over 1300 species from 216 genera (Wallace and Deitz 2004, 2006). Most of the research has focused on the description of new species, and even recently, highly relevant phylogenetic studies have been carried out which have allowed us to better understand the evolution of the centrotines. However, knowledge of immature stages and biological habits is very scarce.

The genus *Campylocentrus* contains 13 species, of which four are reported for Costa Rica: *Campylocentruscurvidens* (Fairmaire), *C.hamifer* (Fairmaire), *C.nigris* Funkhouser, and *C.pusillus* (Fairmaire) (Dmitriev et al. 2024). Two others, *C.brevicornis* Fowler and *C.cavipennis* (Fowler) have been recorded from both Guatemala and Panama, so they probably also occur in Costa Rica (Fowler 1896). These treehoppers are characterized by being robust and vary from brown to black in color; their head is wider than high; the pronotum is convex with strong suprahumeral horns and strong, triangular, blunt humeral angles (Figs 2–4); the posterior process is long and slender with a large ventral lobe that touches the scutellum (diagnostic for the genus) and extends well beyond the internal angles of the forewings and is apically acute (Godoy et al. 2006).

Until now, only the centrotine immatures of the Old World have been illustrated and/or described (Capener 1962, 1968; Ananthasubramanian and Ananthakrishnan 1975a, 1975b; Ananthasubramanian 1978, 1980a, 1980b, 1982; Yuan and Chou 2002).

## ﻿Materials and methods

Morphological terms follow McKamey et al. (2015). Plant classification and authors agree with the World Flora Online (https://www.worldfloraonline.org) and Zuchowski (2022). Photos were taken using an Olympus E-330 camera. After photographing the individual “slices”, they were compiled into a single composite image using the image processing software CombineZP v. 1.0. The stacked images were enhanced and edited using the open-source software GIMP v. 2.10.32. Measurements were carried out using the open-source software ImageJ for Windows 64-Bit.

Voucher specimens are located in the following institutions.

**MIUCR** Museo de Insectos de la Universidad de Costa Rica, San José, San Pedro, Costa Rica

**USNM** National Museum of Natural History, Smithsonian Institution, Washington, DC, USA

## ﻿Results

The description of *Campylocentrus* nymphs must be understood within the context of features of its higher taxa, which are described below. There are six tribes of Centrotinae in the New World: Boocerini, Centrodontini, Monobelini, Nessorhinini, Platycentrini, and Pieltainellini (Wallace and Deitz 2004). Nymphs of the first five tribes have been examined by the second author, which together with the boocerine genus *Ischnocentrus* Stål will be described separately. Nevertheless, the features of these other taxa, and features of other New World tribes and subfamilies, both described and undescribed, have been taken into account in constructing the descriptions of New World Centrotinae and the tribe Boocerini.

### ﻿Subfamily Centrotinae of the New World

**Nymphal description.** Overall body. Waxlike substance absent from body; dorsal contour of abdomen linear in lateral view (Fig. 5); elongate in dorsal view; dark longitudinal lines absent from body. Head. Chalazal bases tuberculate; without enlarged chalazae between eyes; without single enlarged chalaza behind eye or paired crescent-shaped callosity near midline; compound eye posteriorly straight, not emarginate; eye greatly bulging laterally, ventromedial angle convex, considerable portion curved ventrally; without paired rounded protuberances between eyes; dorsal margin not extending over portion of eye. Prothorax. Dorsal pronotal single medial horn bud absent; metopidial sulcus not incised; posterior extension narrowly convex in dorsal view; pronotal humeral horn buds absent; without 3 spines apically; without median carina. Mesothorax. Without clearly delimited subcircular depressed callosity above wingpad and adjacent to pronotal margin; without median carina. Legs. Lateral margins clearly defined; mesothoracic coxa without laterally projecting process. Abdominal terga III–VIII. Scoli absent. Abdominal segment IX. Dorsal structures absent from apex (except consisting of paired enlarged chalazae, directed posteriorly, in some *Ischnocentrus*); position of fused portion distal to unfused portion; unfused portion not bifurcate; ventrolateral margin with paired row of enlarged chalazae.

#### ﻿Tribe Boocerini

Taxon classificationAnimaliaHemipteraMembracidae

Goding, 1892

EC187349-D47E-59DB-9110-3C9ECA3E7BE7

##### Nymphal description.

Overall body. Weakly vertically depressed. Head. Mid-dorsal structures and dorsal or anterior rounded protuberances, and compound eye surface setae absent; chalazal setae narrowly paleate; without enlarged chalazae in front of ventral margin of eye; frons extended over central margin of eye (Fig. 6); not anteriorly or ventrally strongly carinate. Prothorax. Pre- and postmetopidium dorsal structures absent; posterior extension of pronotum not surpassing anterior margin of metanotum; pronotal lateral margin bearing a sharp ventral angle; postmetopidium not elevated and acute; without lateral scoli. Mesothorax. Dorsal structures absent; forewing pad distal two-thirds costal margin emarginate (Fig. 5), costal enlarged chalazae absent; posterior margin convex, medially extending into acute or obtuse angle (Fig. 7); lateral scoli absent. Metathorax. Dorsal structures absent. Legs. Metathoracic tarsi distinctly longer than pro- and mesothoracic tarsi (Fig. 5). Abdominal terga III–VIII. Ventrolateral margins with flattened lamellar extensions on segments IV–VIII (Figs 5, 7); lamellar lateral margins subparallel, apex rounded, chalazae on margins only; lateral scoli absent. Abdominal segment IX. Relative dorsal length subequal to combined lengths of segments VII and VIII; dorsal structures before apex consisting of irregular chalazae; ventral extension subequal to dorsal extension.

#### ﻿ Campylocentrus nigris

Taxon classificationAnimaliaHemipteraMembracidae

Funkhouser

94120675-EDDE-5433-AF51-BD9103FD4D4B

Campylocentrus nigris Funkhouser, 1930: 410.

##### Description.

Adults: length of male 8.4 mm; of female, 9.1 mm. Fifth instar length 7.8 mm. Overall body. Chalazae sparse, almost absent on thorax and abdomen. Head. Without enlarged chalazae adjacent to central margin of eye. Prothorax. Suprahumeral horn buds present high on pronotum (Fig. 5). Mesothorax. Forewing pad surface chalazae sparse with short, weakly paleate, needle-like setae; no lateral row of enlarged chalazae extending onto meso- or metathorax from abdomen. Legs. Chalazae of tibia on anterior and posterior lateral margins, absent or very few on dorsal surface (Fig. 5); prothoracic tibia lateral margin extended; metathoracic first tarsomere longer than pro- and mesothoracic first tarsomeres, but less than half the length of metathoracic second tarsomere. Abdominal terga III–VIII. Lateral rows of enlarged chalazae not manifested; dorsal structures absent from all terga (Figs 5, 7). Abdominal segment IX. Distal half in cross-section strongly vertically depressed; fused portion with strong lamellae laterally.

##### Previously reported records.

• **Costa Rica.** without mentioning a specific province, canton, or district. W.D. Funkhouser. Type, 1 ♂; paratype, 1 ♀ (Funkhouser Collection) from University of Kentucky.

##### Examined material.

**Figure 1.**
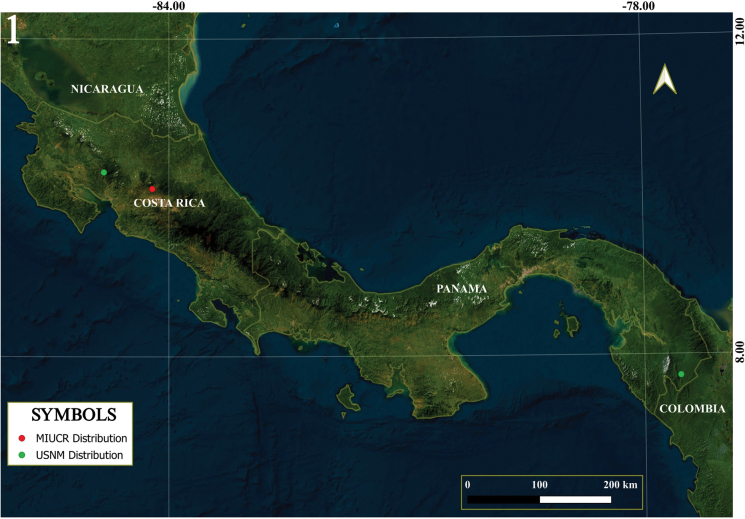
Distributional map of *Campylocentrusnigris* in Costa Rica and Panama.

**New records, (Fig. 1).***Campylocentrusnigris*. • **Costa Rica.** Alajuela, Poás, Sabana Redonda, La Altura. 1480 m. Peraza, A. 31-VII-2024. (3 adults) 2 ♂; 1 ♀; 1 nymph (MIUCR); Puntarenas, Santa Elena, Monteverde. 1440 m. S. McKamey. 18-IV-1984. (10 adults) 6 ♂; 4 ♀; 1 nymph (USNM). Host at both locations: *Sicyosedulis* Jacq. (Cucurbitaceae). **Panama** (**new country record**) Darien Prov., 10 km S El Real, Rio Pirre. 7–18-VI-1984. S. McKamey (24 adults) 10 ♂, 14 ♀, 1 nymph (USNM). Note: The single observation of the species in iNaturalist, from Ecuador, is probably a misidentification.

##### Biological notes.

Host plants associated with this genus. *Campylocentruscurvidens* has been collected on *Glycine* spp. (Fabaceae); *C.hamifer* is associated with *Coffeaarabica* L. (Rubiaceae), *Petiveriaalliacea* L. (Petiveriaceae), *Gonolobusedulis* Hemsl. (Apocynaceae), *Spondiasmombin* L. (Anacardiaceae), *Sicyosedulis* Jacq. (Cucurbitaceae), *Iochromaarborescens* (L.) J.M.H. Shaw (Solanaceae) and plants from the family Anacardiaceae; *C.nigris* has been recorded on plants from the family Asteraceae, and *C.pusillus* on *Chromolaenacorymbosa* (Aubl.) R.M.King & H.Rob. (Asteraceae), *Ipomoeapurga* (Wender.) Hayne, *Ipomoeatiliacea* (Willd.) Choisy (Convolvulaceae), *Medicagosativa* L. (Fabaceae), *Cucurbitaficifolia* Bouché, *Cucurbitapepo* L. (Cucurbitaceae), *Passifloraligularis* Juss. (Passifloraceae) and *Zeamays* L. (Poaceae) (Ballou 1935, 1936, 1937; Godoy et al. 2006). *Campylocentrusnigris* (as *Campylocentrus* sp.) has been recorded from *Sicyos* sp. (as *Sechium* sp.) (Godoy et al. 2006, Figs 8–10).

**Figures 2–4.**
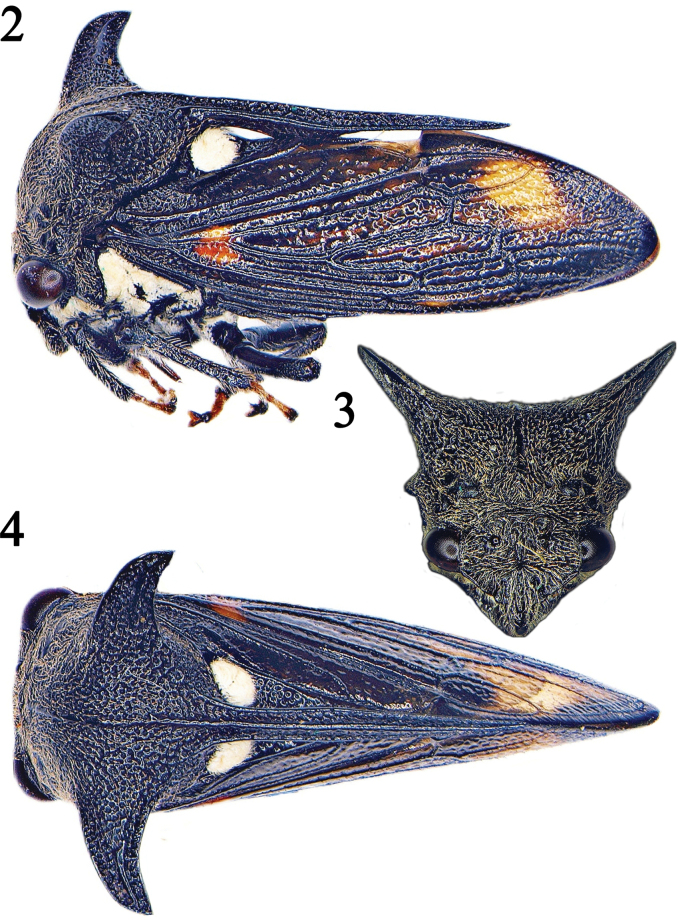
*Campylocentrusnigris* male. **1** Lateral view; **2** Frontal view of head and pronotum; **3** Dorsal view; **4** Lateral view.

##### Behavior.

In escape response to a stalking predator of Neotropical true hoppers (Hemiptera suborder Auchenorrhyncha) Galatowitsch and Mumme (2004) reported that the defense of *Campylocentrus* is structural, and its jumping response when approached by its predator was the most sensitive true hopper studied, with an average jump distance of 21.4 cm when approached by its predator.

**Figures 5–7.**
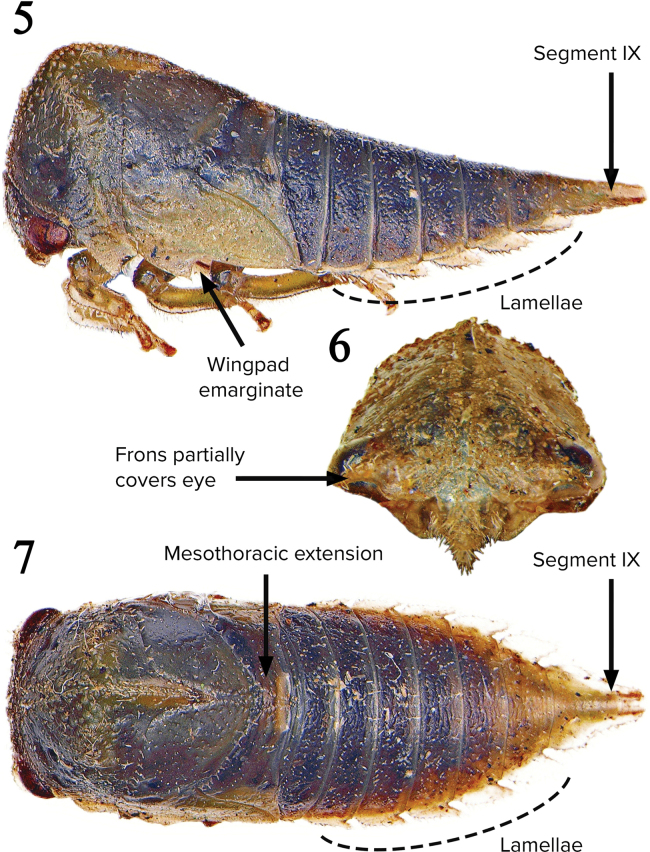
Last instar of *Campylocentrusnigris*. **5** Frontal view of head and pronotum; **6** Dorsal view; **7** Lateral view.

**Figures 8–10.**
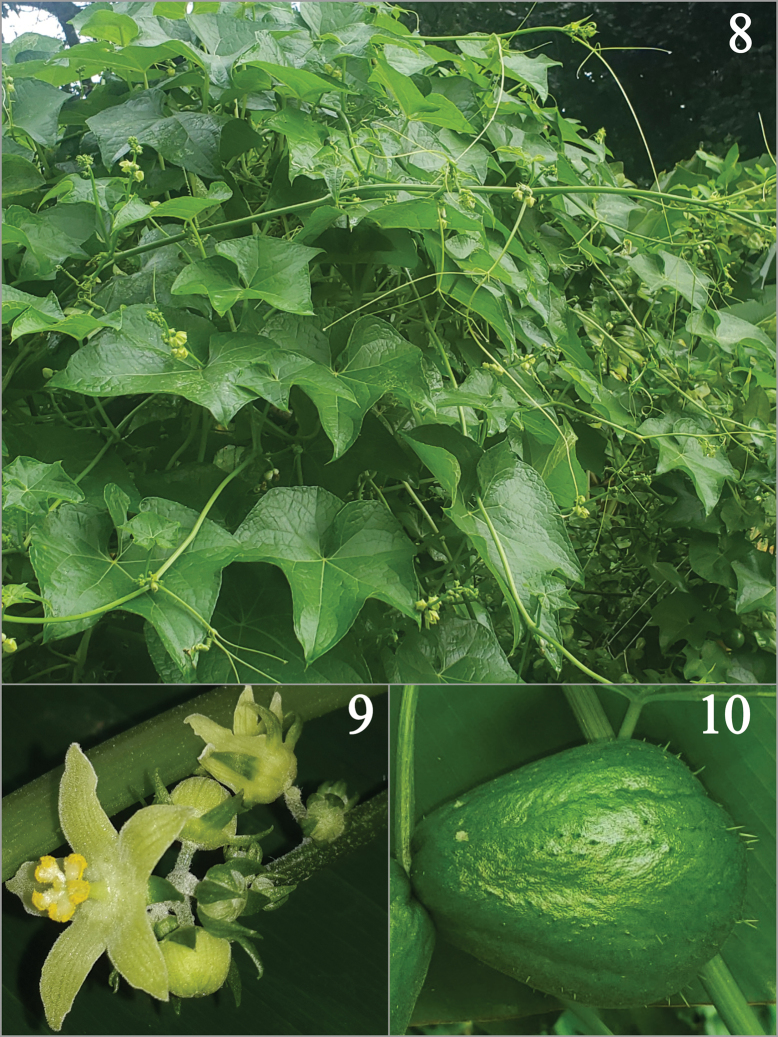
Host plant *Sicyosedulis* (Cucurbitaceae), commonly named vegetable pear (English) and chayote (Spanish). **8** Plant; **9** Flower; **10** Fruit..

**Figures 11–16.**
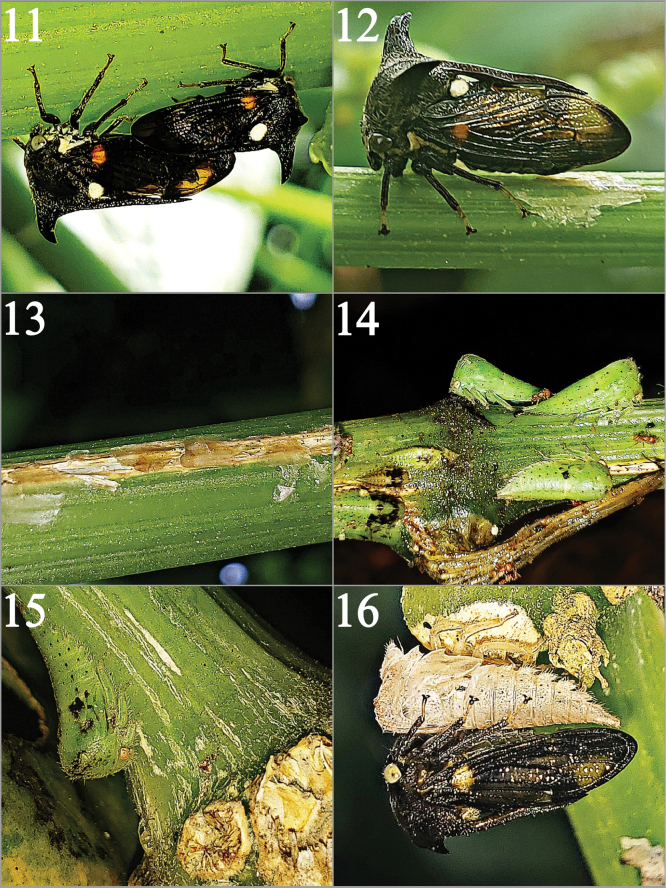
Live photographs of *Campylocentrusnigris* in situ without manipulation. **11** Copulation on stem left female, right male; **12** Females cover the ovipositional slits with secretions; **13** Ovipositional secretions after a week; **14** Immature stages being attended by ant (Formicidae); **15** Cryptic nymph on the node of a mature stem; **16** Recently molted adult next to its exuvia.

In Costa Rica it is common to call “chayotera” the established cultivation of “chayote” or a plant spread in yard or vacant lots the specimens observed here fed on that (Figs 8–10). The eggs are deposited and embedded in the stems, later the females cover the openings made with secretions, making a kind of suture (Figs 12, 13). The nymphs observed were in groups; however, the last instars tended to separate and locate themselves in the nodes of the Cucurbitaceae stems. In addition, the resting position of the tibiae enhances their crypsis; the prothoracic tibia resting against the anterior margin of the pronotal postocular lobe (*pol*), the mesothoracic tibia resting against the truncate posterior margin of the *pol*, and the metathoracic tibia fitting into the emarginate notch of the wingpad. The result is that there is no space between the ventral contour of the nymph and the plant surface. The abdominal lamellae further obfuscate the insect’s outline. The crypsis they achieve by positioning themselves in this way is notable, it practically seems like just another knot (Fig. 15). It was also observed that the last instars seek to position themselves under the stems or tendrils for the emergence of the adult (Fig. 16). The nymphs were sometimes attended by ants (Fig. 14) (Formicidae-Myrmicinae). Adults were active in the morning hours from 7:00 a.m. to 11:00 a.m. and in the afternoon from 12:00 p.m. to 2:00 p.m., showing short but very fast flights. Adults most commonly were located on the stems and, rarely, on the undersides of leaves. During this period, both courtship and copulation occur (Fig. 11).

## Supplementary Material

XML Treatment for Boocerini

XML Treatment for Campylocentrus nigris

## ﻿Additional information

### Author ORCIDs

Andrey J. Peraza-Sánchez https://orcid.org/0000-0002-1940-3673

Stuart H. McKamey https://orcid.org/0000-0002-9617-0594

### Data availability

All of the data that support the findings of this study are available in the main text.
